# Unexpected consequences: exon skipping caused by CRISPR-generated mutations

**DOI:** 10.1186/s13059-017-1240-0

**Published:** 2017-06-14

**Authors:** Joshua J. Sharpe, Thomas A. Cooper

**Affiliations:** 1 Department of Pathology and Immunology, Baylor College of Medicine, Houston, TX 77030 USA; 2 Department of Molecular and Cellular Biology, Baylor College of Medicine, Houston, TX 77030 USA; 3 Department of Molecular Physiology and Biophysics, Baylor College of Medicine, Houston, TX 77030 USA

## Abstract

A new study finds that splicing disruption is a frequent consequence of mutations generated by CRISPR/Cas9 gene-editing technology, and alleles designed to be null can express aberrant proteins. This new information allows enhanced quality control procedures to select the best mutant alleles generated by CRISPR/Cas9.

Please see related Method article: https://www.doi.org/10.1186/s13059-017-1237-8

## Introduction

A common application of CRISPR/Cas9 (clustered regularly interspaced short palindromic repeats/CRISPR-associated system 9) gene-editing technology (commonly referred to as “CRISPR” for short) is to use an individual single-guide RNA (sgRNA) construct to introduce double-stranded breaks within coding exons. This activates DNA repair by nonhomologous end-joining (NHEJ) and introduces insertions or deletions (indels) of a small number of nucleotides [1]. Indels that are not a multiple of three nucleotides shift the reading frame and introduce premature termination codons (PTCs), resulting in mRNA degradation by nonsense-mediated decay (NMD) [2].

This approach is used to generate clonal cell lines and genetically modified organisms with a null mutation of the targeted gene. An unintended consequence, however, is that sgRNA can introduce double-stranded breaks at non-targeted sites within the genome. The potential for these off-target effects are well known; thus, procedures are in place to reduce their frequency, screen for their occurrence, and in the case of sexually reproducing organisms, perform outcrossings for their exclusion from lines with the desired mutation.

A paper by Mou et al. in this issue of *Genome Biology* [3], a report by Kapahnke et al. [4], and a recent result in zebrafish [5] have identified exon skipping as a new and relatively frequent unintended consequence of CRISPR-generated mutations. While exon skipping is not a problem if the resulting mRNA(s) is (are) subject to NMD, it could produce mRNAs that express an aberrant protein rather than the intended null allele. The results reveal the potential for CRISPR-generated cell lines and organisms to produce artifactual effects. The good news is that just as for off-target effects, awareness of the problem allows better screening for truly null alleles.

## CRISPR-generated mutations can cause exon skipping

**Fig. 1:**
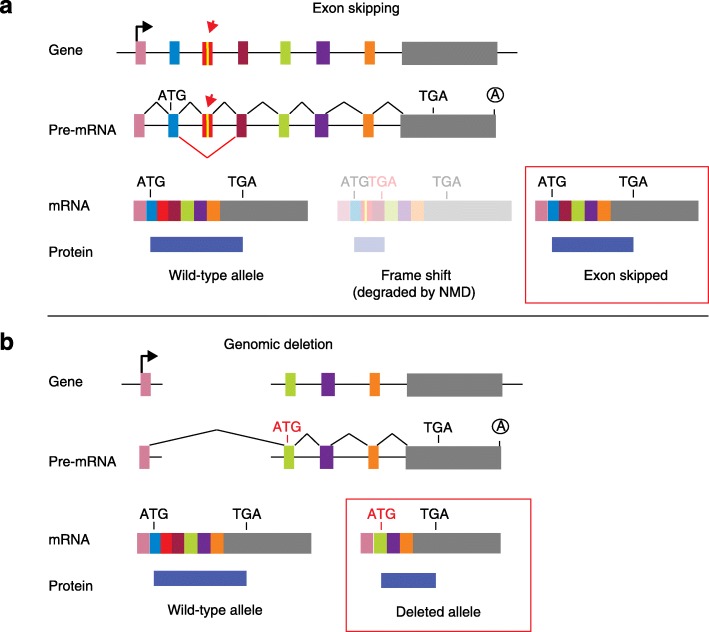
Two mechanisms for exon skipping. **a** Clustered regularly interspaced short palindromic repeat (CRISPR)-induced indel (*red arrow*) results in the intended mRNA with a premature termination codon subject to nonsense-mediated decay (*NMD*), but skipping the mutated exon retains the reading frame and produces an aberrant protein. **b** CRISPR-induced genomic deletion removes three exons, including the translation initiation codon, such that a downstream internal ATG produces a protein that is truncated at the N-terminus. *Red boxes* indicate mRNAs that produce aberrant proteins

Mu et al. found that targeting exons using CRISPR and a single sgRNA in cell lines produced exon skipping by two mechanisms that appear to be independent (Fig. 1). The first occurs during splicing of the mutated pre-mRNA, and the second is caused by genomic deletions that remove multiple exons and splicing of the remaining exons.

The researchers used CRISPR to generate two clonal derivatives of a lung adenocarcinoma cell line, one with a single nucleotide deletion and the other with a two-nucleotide deletion in exon 2 of the Kras oncogene. Both of the mutations introduce a PTC just downstream of the translation start codon. Typically NMD is more efficient when the PTC is toward the 5′ end of the mRNA [2], so a very low level of mRNA from the mutated allele was expected. However, RNA sequencing (RNA-seq) data from the cell lines revealed weak knockdown levels of the transcript, fewer exon 2 reads compared to exons 1 and 3, and junction reads indicating splicing of exons 1 and 3 that was not prevalent in wild-type cells. Reverse transcription-PCR (RT-PCR) using PCR primers in exons 1 and 3 showed a substantial level of mRNAs that were missing exon 2 and had no translation start codon. These transcripts would result in an N-terminal truncated protein caused by the initiation of translation at an in-frame ATG in exon 3.

Next, Mu et al. targeted the in-frame exon 3 of the β-catenin gene (*Ctnnb1*). This exon, if skipped, produces a stabilized, constitutively active β-catenin protein that is retained in the nucleus. On testing multiple sgRNAs, many efficiently introduced indels that also produced a fraction of exon-skipping mRNAs, regardless of the strand targeted. Immunoblotting and immunofluorescence staining revealed that the protein product resulting from exon 3-skipping was localized to the nucleus instead of in the cytoplasm, supporting the concern that alleles targeted for a null mutation can produce functional proteins.

But there was more. When primers used for RT-PCR were located multiple exons upstream and downstream of the *Ctnnb1* targeted exon, multiple bands were detected that resulted from skipping not just the targeted exon, but also flanking exons. The explanation is that CRISPR-induced large genomic deletions that removed these exons, and the remaining exons were spliced. At first, this deletion was not detected by genomic DNA PCR since the binding sites for primers designed to detect small indels within the exon were removed by the deletion.

The results by Mou et al. [3] provide the clearest evidence to date that exon skipping occurs at relatively high frequency in mRNAs from CRISPR-generated alleles. They also show that potentially undetected genomic deletions can lead to exon skipping. Finally, they demonstrate that exon skipping results in the expression of a truncated protein with different localization, and presumably a different function, from the wild-type protein. The message is that whether a single exon is skipped because of a small indel, or multiple exons are skipped because of a genomic deletion, an allele targeted for a null mutation can produce a protein with residual normal activity or a new gain-of-function.

## What is the mechanism?

The mechanism for skipping multiple exons is straightforward: the exons that remain intact after a genomic deletion are spliced. More complex and concerning is the fact that a change of only one to a few nucleotides can result in exon skipping during pre-mRNA splicing. It has been known for some time that, in addition to the splice sites at the intron–exon boundaries, exons contain sequences that act positively or negatively on splicing efficiency [6]. Positive-acting elements within exons, known as exon splicing enhancers (ESEs), bind factors that enhance recognition by the splicing machinery. Negative-acting elements (exon splicing silencers (ESSs)) are thought to prevent the use of cryptic splice sites. One can hypothesize that an indel promotes exon skipping either by disrupting an ESE or by fortuitously introducing an ESS. Such effects are not as unlikely as one might think. A growing number of examples of genetic variants produce differences in splicing efficiency between individuals. Up to 30% of disease-causing point mutations do so by disrupting splicing, and half of these are outside of consensus splice sites—most often within exons [7]. Consistent with a partial loss of ESE function (or a gain of weak ESS activity), exon skipping was partial, and often a relatively small fraction of mRNAs lacked the skipped exons. Still, the frequency with which small CRISPR-induced indels produced a change in splicing is surprising.

In addition to the multiple *cis*-acting elements within and around exons, differences in the nuclear *trans*-acting environment also play a role in splicing efficiency [6]. A mutated exon might show some level of skipping in one cell line and none in another. In a genetically modified organism, tissue-specific differences in the level of exon skipping might produce tissue-specific differences in the expression of an aberrant protein product.

## What is one to do?

Ideally, it would be possible to know a priori how to target a gene and avoid exon-skipping issues. However, it is currently difficult to predict the effect of a given indel on splicing efficiency based on exon sequence. Algorithms to identify exonic splicing elements have been partially successful, and computational definition of the so-called splicing code is ongoing [8–10], but these cannot yet completely predict the effect of a given nucleotide change on splicing efficiency.

The good news is that awareness of the problem leads to enhanced quality control. First, it is recommended to use RT-PCR to determine whether the exon containing the indel is skipped, and if so, to determine the protein-coding potential. Second, a single sgRNA can produce a large deletion, and this might remain undetected because the priming sites for at least one primer designed for PCR of an expected smaller deletion might be lost. Therefore, one needs to account for the structure of both alleles (or more if the cell is polyploid for the targeted chromosome), since one allele might contain an indel but the other might contain a large undetected deletion. An RT-PCR survey of the mRNA is a straightforward screen to identify mRNAs lacking exons generated by either mechanism. For example, RT-PCR using primers located several exons upstream and downstream of the targeted exon—or even in the first and last exons—will readily detect an mRNA produced by splicing the remaining exons of a large deletion. As a first pass screen, RT-PCR can more rapidly indicate a deletion compared to a PCR screen of genomic DNA for unknown deletion endpoints.

CRISPR is straightforward, inexpensive, and widely accessible to individual laboratories. As well as establishing rigorous protocols for highly efficient and selective mutagenesis, it is important to set up rigorous quality controls to ensure that the mutants created do not contain hidden surprises that could produce artifacts.

## Abbreviations

Cas9: CRISPR-associated system 9
CRISPR: Clustered regularly interspaced short palindromic repeats
ESE: Exon splicing enhancer
ESS: Exon splicing silencer
indel: Insertion or deletion mutation
NHEJ: Non-homologous end-joining
NMD: Nonsense-mediated decay
PTC: Premature termination codon
RT-PCR: Reverse transcription polymerase chain reaction
sgRNA: Single-guide ribonucleic acid
